# Coronary Artery Disease in Systemic Lupus Erythematosus: What Do the Facts Say?

**DOI:** 10.7759/cureus.33449

**Published:** 2023-01-06

**Authors:** Efrén Melano-Carranza, Alexis Zambrano-Zambrano, Walter Valle-Uitzil, Alejandro Ezquerra-Osorio, Axel Rodriguez-Méndez, Juan H Larios-Lara, Luis Baeza, Juan Andres Pimentel-Esparza, Jorge Antonio Cervantes-Nieto, Juan Alan Fuentes Mendoza

**Affiliations:** 1 Cardiac Intensive Care Unit, National Institute of Cardiology, Mexico City, MEX; 2 Internal Medicine, National Medical Center November 20 ISSSTE, Mexico City, MEX; 3 Internal Medicine, ABC Medical Center, Mexico City, MEX; 4 Internal Medicine, PEMEX Regional Hospital Salamanca, Salamanca, MEX; 5 Cardiology, PEMEX Regional Hospital Salamanca, Salamanca, MEX; 6 Cardiology, Policlínica Integral del Bajío, Irapuato, MEX

**Keywords:** cardiovascular risk assessment, pathogenesis, coronary artery disease, cardiovascular disease, systemic lupus erythematosus

## Abstract

Systemic lupus erythematosus (SLE) is an autoimmune disease that can affect any organ with a predisposition for women of reproductive age. It is related to a higher risk of cardiovascular events, increasing it up to 50 times in young people, and 30% of deaths are attributed to coronary artery disease. The risk of developing cardiovascular disease in SLE is related not only to traditional cardiovascular risks factors such as advanced age, hypertension, dyslipidemia, and diabetes but also to disease-specific factors, such as degree of activity, autoantibodies, organ damage, and treatment. Accelerated atherosclerosis is one of the main contributors to pathogenesis. Manifestations range from angina to acute myocardial infarction and sudden death. Markers have been studied for the detection of subclinical disease and stratification of these patients, as well as different treatment options to improve the cardiovascular prognosis of the disease.

## Introduction and background

Systemic lupus erythematosus (SLE) is an inflammatory autoimmune disease with heterogeneous systemic manifestations characterized by exacerbations and remissions. It has a prevalence of 50-70 cases and an incidence of one case per 100,000 people per year [1]. Women of reproductive age between 15 and 44 years of age are the population most predisposed to developing SLE. The female/male ratio is reported in the different cohorts up to 13:1 [2].

Cardiovascular disease represents one of the leading causes of death in patients with SLE [3]. It is related to a higher risk of adverse events such as coronary heart disease, stroke, and peripheral arterial disease [2]. An approximately two-fold increased risk has been observed for cardiovascular events and acute myocardial infarction (AMI), a five-fold increased risk, and earlier age of presentation compared to controls, 61 to 63 years, depending on the cohort [4].

About 30% of SLE patients' deaths are attributed to coronary artery disease (CAD). It has been seen that premenopausal women with SLE have up to a 50-fold higher risk of AMI when compared to healthy women of the same age, with the first year of diagnosis being the most frequent presentation [1]. Most acute coronary syndrome (ACS) cases in patients with SLE are due to atherosclerosis, which in post-mortem studies has been found in 50% of patients [5]. Less common causes include thrombosis, vasculitis, and spontaneous coronary artery dissection.

A study in Sweden that included 34 patients with AMI and SLE compared to another with AMI without SLE identified a significantly greater number of diseased vessels. Statistically insignificant, it was documented that the most frequent diseased vessel was the anterior descending artery in 73% vs. 59%, obstructive CAD was the etiology in 88% vs. 66%, and AMI with ST-segment elevation presented a similar frequency [4,6].

## Review

Pathogenesis

**Figure 1:**
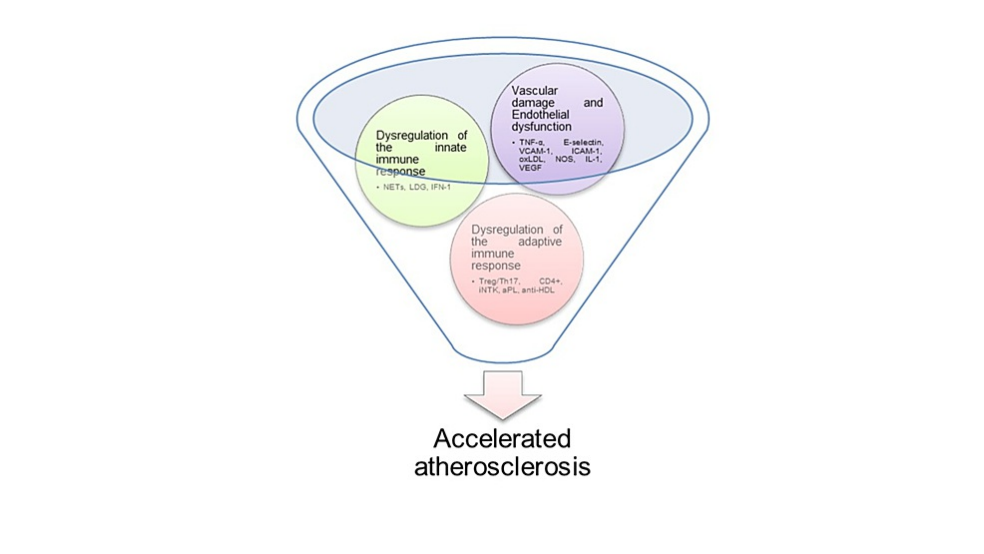
Vascular damage in SLE SLE: systemic lupus erythematosus; TNF- α: tumor necrosis factor-alpha; VCAM-1: vascular cell adhesion molecule 1; ICAM-1: intercellular adhesion molecule 1; IL-1: interleukin 1: VEGF: vascular endothelial growth factor: oxLDL: oxidized LDL; LDG: low-density granulocytes; NETs: neutrophil extracellular traps; Treg: regulatory T cell; Th17: T helper 17 cells; CD4+: cluster of differentiation 4; iNTK: invariant natural killer T; aPL: antiphospholipid antibodies; anti-HDL: anti-high-density lipoprotein

The specific mechanisms in SLE that contribute to cardiovascular disease are still under investigation. The main contributors are documented accelerated atherosclerosis, coronary vasculitis, thrombotic events, vasospasm, embolization phenomena, and hypertensive remodeling [7]. Accelerated atherosclerosis appears to be the main factor resulting from immune-mediated inflammation. Atherosclerotic plaque formation in SLE patients is characterized by three main phenomena (Figure 1).

Vascular Damage and Endothelial Dysfunction

Microvascular damage and endothelial dysfunction have been observed in the early stages of SLE, even without cardiovascular risk factors. It provokes an inflammatory response to its repair. In chronic injuries, this response deregulation is due to a decrease in endothelial progenitor cells and repair cell dysfunction, resulting in low levels of vascular endothelial growth factor (VEGF) that cause the poor repair and promote plaque formation [2,8,9]. Tumor necrosis factor-alpha (TNF-α) and interleukin 1 (IL-1) recruit T cells and monocytes that, in contact with the endothelium, express adhesion molecules (E-selectin, vascular cell adhesion molecule 1 (VCAM-1), and intercellular adhesion molecule 1 (ICAM-1)), and their increase is linked to higher coronary calcium scores in LES2; they also produce reactive oxygen species that, when interacting with low-density lipoprotein (LDL), form oxidized LDL (oxLDL). Endothelial cells exposed to oxLDL form monocyte chemotactic protein-1 (MCP-1) and other chemokines that stimulate the differentiation of macrophages to foam cells. Foam cells in smooth muscle form fibrosis and atherosclerotic plaques [5]. There is a decrease in the enzyme nitric oxide synthase (NOS) due to endothelial dysfunction, causing a reduction in the production of nitric oxide [2]. Abnormal coronary flow has been documented in patients with normal coronary arteries in positron emission tomography (PET) patients [10].

Dysregulation of the Innate Immune Response

Toll-like receptors (TLR) 7 and 9 present in macrophages, dendritic cells, and neutrophils activate the transcription of type I interferons (IFN-1) α and β, which are an important marker of activity in SLE. Their elevation suppresses the production of endothelial progenitor cells and activates macrophages for the formation of foam cells. TLR 7 and 9 are associated also with platelet activation and thrombosis. Low-density granulocytes (LDG) are a subtype of neutrophils with a greater capacity to form neutrophil extracellular traps (NETs) in patients with SLE that promote thrombus formation and activation of autoimmune responses against nuclear antigens [2,8,9].

Dysregulation of the Adaptive Immune Response

The presence of autoantibodies against endothelial cells and a more significant number of apoptotic cells suggests an autoimmune response. The impaired ability to remove apoptotic cells and debris from oxidized lipoproteins in the arterial wall causes a loss of immune tolerance for various autoantigens [8]. Regulatory T cells suppress atherogenic T cells. In SLE, Treg/Th17 imbalance is common. Th-1 cells produce interferon-gamma (IFN-γ) and inhibit the growth of endothelial cells and smooth muscle in excess, leading to instability of the atherosclerotic plaque. There is also excessive inflammation due to IL-17 produced by Th-17 cells [2,5]. In SLE, CD4+ cells migrate to the arterial wall with proatherogenic activity, and their increase is related to the development of coronary and carotid atherosclerosis [5].

In asymptomatic coronary disease, invariant natural killer T (iNKT) cells have been shown to produce an atheroprotective effect on the plaque by differentiating macrophages toward an anti-inflammatory phenotype. On the contrary, in symptomatic coronary disease, iNKT cells do not have the atheroprotective effect, and proinflammatory monocytes increase [5].

B cells produce autoantibodies. B1 cells create harmful IgG antibodies, a factor related to SLE. Antiphospholipid antibodies (aPL) of the IgG and anti-high-density lipoprotein (anti-HDL) type are independent indicators of plaque development [2]. The increase in Apo-A, decrease in ApoB100, and hyperleptinemia is also related to the progression of atherosclerosis through IL-6, TNF-α, IL-17, and other cytokines [2].

Risk factors

Cardiovascular risk factors are classified into classic or traditional and non-classic factors (such as age, gender, hypertension, smoking, etc.) or those specifically related to SLE.

Traditional risk factors include insulin resistance and diabetes, more frequent in SLE; systemic arterial hypertension, with a prevalence of up to 74% in SLE [10]; smoking and dyslipidemia contribute to coronary calcification, and a pro-inflammatory environment, in turn, is favored by SLE [11]. More significant amounts of dysfunctional HDL (d-HDL) have been found in SLE, and this correlates with the severity of coronary disease [5]. Asymptomatic atherosclerotic disease is higher in SLE, with higher carotid intima-media thickness scores and a 2.5-fold increase in the prevalence of carotid plaques. There is also an increase in visceral adipose tissue and an altered distribution, with greater aortic perivascular density, linked to aortic and coronary calcification [2].

The factors related explicitly to SLE and its pro-inflammatory environment, not yet fully understood in its mechanism and progression of cardiovascular disease, are the prolonged duration of SLE, which is independently associated with the formation of carotid and coronary plaques. Anti-endothelial cell antibodies and antiphospholipid antibodies are related to increased cardiovascular risk; their presence at the start of diagnosis predicted the appearance of cardiovascular events (P = 0.007) according to the Lupus in minorities: nature versus nurture (LUMINA) cohort study [12], although the exact mechanisms are still unknown [10,12]. In addition, positive anti-dsDNA, anti-Ro, and anti-Sm antibodies were associated with a higher risk of cardiovascular disease (P = 0.032, 0.015, and 0.026, respectively) [12]. Kidney damage contributes to the progression of CAD, endothelial damage, arterial stiffness, and plaque formation [10]. Glucocorticoid therapy also increases the risk of developing CAD by contributing to glycemic abnormalities, hypertension, obesity, hyperlipidemia, and its duration of treatment increases the risk of clinical and subclinical cardiovascular diseases. It has been shown that treatment with prednisone, more significant than 30 mg/day, increases the risk when comparing patients with SLE who did not take it [13].

Evaluation and diagnosis

The clinical spectrum of CAD includes manifestations ranging from angina and functional class limitation to AMI and sudden death.

There is no optimal method to predict the cardiovascular risk of patients with SLE in different populations. Cardiovascular risk calculators such as Globo Risk, SCORE, and ASCVD start from the age of 40 years, which is a limitation for calculating the risk in the population with SLE that begins at an earlier age. The risk calculators validated in patients without SLE underestimate the risk [14]. QRISK 3 score (https://qrisk.org/three/) calculates patients with autoimmune diseases such as SLE and corticosteroids to predict AMI and cerebrovascular events at 10 years. Another tool proposed by the Systemic Lupus Erythematosus International Collaborative Clinics (SLICC) and the American College of Rheumatology is the damage index (SDI), which measures cumulative damage during the course of SLE and has been found to predict 10-year mortality (P = 0.0002) with a trend toward more cardiovascular disease (P = 0.056). A damage index ≥ 1 was associated with cardiovascular events (P < 0.001). The predictive role of activity indices such as SELENA-SLEDAI shows controversial results [12].

New serum markers have been proposed to measure the activity of the disease, as evidenced in different studies that indicate that C-reactive protein (CRP) and homocysteine can be helpful markers [1,10]. Antiphospholipid antibodies are independent cardiovascular risk markers already established in different cohorts [10,12]. More recently, osteoprotegerin, leptin, and low levels of vitamin D have been associated with an increased risk of CAD [10]. The role of each one in the development of cardiovascular disease has not yet been established, and its measurement should be included together with other factors.

Studies have shown subclinical alterations through imaging findings, including echocardiography (mitral and tricuspid regurgitation, mitral and aortic thickening, pericardial effusion, and pulmonary arterial hypertension) [15], and magnetic resonance imaging (pericardial effusion, late gadolinium enhancement of a non-ischemic pattern, stress-perfusion deficits) [16,17]. In SLE, subclinical CAD by coronary computed tomography (CT) angiography is more frequent, and there is more multivessel disease; coronary plaque in women was associated with worse clinical results compared to men, and the measurement of coronary calcium by CT can help estimate the residual risk of cardiovascular disease in patients with SLE, particularly in severe initial disease and lupus nephritis, which has recently been associated with higher mortality [18]. Based on this evidence, the authors recommend evaluating patients by employing an imaging study such as determining coronary calcium by tomography from the moment of diagnosis to establish subclinical cardiovascular disease.

On the other hand, although atherosclerosis is the primary cause of coronary disease, vasculitic involvement of the coronary arteries should also be considered, mainly due to therapeutic considerations. Ischemia due to vasculitis occurs more frequently in young patients with a recent disease diagnosis who are in some period of activity [7]. For all of the above, the cardiovascular assessment of patients with SLE should include a general perspective of all classic and non-classic risk factors, as well as the use of validated tools to predict outcomes and be able to treat the disease from its subclinical forms.

Prevention and treatment

There is controversial evidence on the different interventions that seek to reduce cardiovascular risk in patients with SLE.

Management will depend on the presentation of CAD. The acute and chronic coronary syndrome should be managed following the recommendations of international guidelines. In a cohort with 2652 patients with AMI and SLE, it was found that in AMI with ST elevation and without ST elevation, hospital mortality, revascularization strategy, medical treatment, and costs were similar compared to others with AMI without SLE, being only significantly higher percutaneous coronary intervention in non-ST elevation AMI (32.9% vs. 29.6%, OR 1.16 p=0.04) [19].

In the case of subclinical atherosclerotic disease, pharmacological treatment and lifestyle modifications are the goal, which should be more aggressive based on current evidence of accelerated atherosclerosis in SLE.

Non-pharmacological measures include stopping smoking, glycemic control with low glycemic index and hypocaloric diets, contributing to weight loss, and decreasing abdominal circumference [20]. The supplementation of omega-three acids in the diet has not shown benefit in improving endothelial dysfunction or disease activity [21]. Supervised physical activity enhances the quality of life and aerobic capacity and improves endothelial function [22].

Statins have been proposed for their lipid-lowering, anti-inflammatory, and immunomodulatory effects; however, different clinical trials and observational studies show contradictory evidence of their benefits [7,10,11,13]. The evidence suggests that atorvastatin has not been proven to reduce the progression of atherosclerosis in patients with SLE according to Lupus Atherosclerosis Prevention Study (LAPS) [10]. Rosuvastatin and fluvastatin have been shown to reduce indirect markers of atherosclerotic disease progression in specific subgroups of patients; Rosuvastatin showed decreased levels of high-sensitivity CRP and thrombomodulin [23], compared to fluvastatin which demonstrated a reduction LDL cholesterol and with a 73.4% reduction in the frequency of major cardiac events [24].

Strict blood pressure control is mandatory. There is no specific pharmacological therapy to reduce blood pressure figures, except in patients with lupus kidney disease, where angiotensin-converting enzyme inhibitors (ACEI) are recommended, using in the rest of the patients the recommendations for the general population [11].

The use of acetylsalicylic acid as primary prevention in patients with SLE has been tested in different clinical trials and meta-analyses, showing a decrease in thrombotic events and cardiovascular events, including a positive and negative population for antiphospholipid antibodies [25,26]. Some experts recommend a low dose in patients with a diagnosis of SLE unless there is a contraindication [27]; however, the European Alliance of Rheumatology Associations (EULAR), in its publication on cardiovascular risk, mentions adding acetylsalicylic acid with a base to cardiovascular risk without mentioning it in the risk scale used [28]. There are no recommendations for anticoagulant therapy in patients with SLE unless an indication warrants it.

Therapy directed against SLE activity with hydroxychloroquine has proven to be effective in the primary prevention of thrombotic events in patients with SLE and cardiovascular risk factors by playing a role in lowering cholesterol, inhibiting platelet aggregation, better glycemic control, and insulin resistance [2,5,11]. However, the effect seems to depend on the treatment time with the drug [29].

Research into treatment for cardiovascular prevention in patients with SLE is still under study, evaluating drugs with different mechanisms of action, such as belimumab, mechanistic target of rapamycin (mTOR) inhibitors, and proprotein convertase subtilisin-kexin type 9 (PCSK9) inhibitors [11].

**Figure 2:**
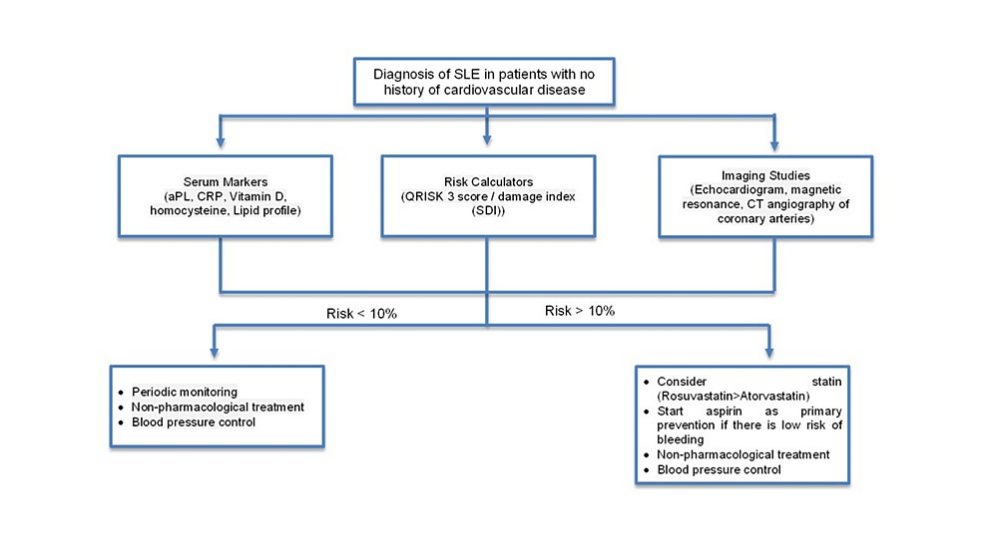
Proposed algorithm for cardiovascular risk assessment in patients with SLE SLE: systemic lupus erythematosus; aPL: antiphospholipid antibodies; CRP: C-reactive protein; CT: computed tomography

The selection of pharmacological and non-pharmacological therapy in patients with SLE to reduce cardiovascular risk should include a comprehensive evaluation with serum tests (LDL, CRP, homocysteine, antiphospholipid antibodies, etc.), imaging tests (coronary CT angiography), and validated risk calculators to achieve a complete change in the patient's lifestyle. We recommended always maintaining the patient with hydroxychloroquine if there is no contraindication. For the start of statin and acetylsalicylic acid in primary prevention, we recommend using cardiovascular risk scales when age allows, such as Globo Risk, which includes the Mexican population, plus a scale validated in the population with SLE, such as the QRISK 3 score. Consideration should be given to relying on other tools, such as calcium score by CT angiography and CRP, to determine the start of primary prevention when the scales are low risk or cannot be determined (Table 1 and Figure 2).

**Table 1:** Cardiovascular risk factors and current treatment goals ACEI: angiotensin-converting enzyme inhibitors; ARA II: angiotensin-II receptor antagonists; LDL: low-density lipoprotein; SGLT-2i: sodium-glucose cotransporter-2 inhibitor; GLP-1 RA: Glucagon-like peptide-1 receptor agonists

Cardiovascular Risk Factor	Treatment Goals
Systemic Arterial Hypertension	Target blood pressure <130/80 mmHg. ACEIs/ARA II as the first line in patients with lupus nephritis or protein/creatinine ratio >500 mg/g.
Dyslipidemia	Dietary management. Maintain LDL < 100 mg/dl (Optional < 70 mg/dl), non-HDL < 130 mg/dl. Consider high-intensity statin.
Diabetes mellitus/Insulin resistance	Glycemic control with the recommendations of the general population. Consider drugs with cardiovascular benefit as first-line (SGLT-2i and GLP-1 RA).
Obesity	Weight loss. Management with the recommendations of the general population.
Inflammation/Disease Activity	Consider hydroxychloroquine. Consider statin (Rosuvastatin > Atorvastatin). Consider aspirin in high cardiovascular risk as primary prevention and the absence of contraindications.
Glucocorticoid treatment	Lowest effective dose. Consider dose < 7.5 mg of prednisone or equivalent.

## Conclusions

SLE is associated with an increased risk of accelerated atherosclerosis and cardiovascular events, which can occur early and late in the disease. There is a strong relationship between specific pathophysiological processes of SLE and classic risk factors. Activity markers and imaging studies can be used to assess the risk and subclinical detection of the disease. In patients with SLE, traditional risk factors should be treated according to the general guidelines for cardiovascular prevention. Hydroxychloroquine has shown efficacy in preventing thrombotic events, and the minimum dose of glucocorticoids should always be considered. Statins have shown protective effects on the vascular wall, but the results have been inconsistent in recommending them to all patients with SLE. Acetylsalicylic acid has demonstrated benefits in the presence of cardiovascular risk. The poor understanding of the mechanisms responsible for cardiovascular complications in SLE is a limiting factor in developing effective preventive therapies. Autoimmunity-related vascular injury may be one of the mechanisms that may be the target of future treatments.
